# The Complexity of Targeting PI3K-Akt-mTOR Signalling in Human Acute Myeloid Leukaemia: The Importance of Leukemic Cell Heterogeneity, Neighbouring Mesenchymal Stem Cells and Immunocompetent Cells

**DOI:** 10.3390/molecules21111512

**Published:** 2016-11-11

**Authors:** Annette K. Brenner, Tor Henrik Andersson Tvedt, Øystein Bruserud

**Affiliations:** 1 Section for Haematology, Department of Clinical Science, University of Bergen, 5021 Bergen, Norway; annette.brenner@uib.no (A.K.B.); tor.henrik.anderson.tvedt@helse-bergen.no (T.H.A.T.); 2 Department of Medicine, Haukeland University Hospital, 5021 Bergen, Norway

**Keywords:** acute myeloid leukaemia, mesenchymal stem cells, therapy, stem cell niche, PI3K-Akt-mTOR, monocytes, membrane molecules, cytokine release

## Abstract

Therapeutic targeting of PI3K-Akt-mTOR is considered a possible strategy in human acute myeloid leukaemia (AML); the most important rationale being the proapoptotic and antiproliferative effects of direct PI3K/mTOR inhibition observed in experimental studies of human AML cells. However, AML is a heterogeneous disease and these effects caused by direct pathway inhibition in the leukemic cells are observed only for a subset of patients. Furthermore, the final effect of PI3K-Akt-mTOR inhibition is modulated by indirect effects, i.e., treatment effects on AML-supporting non-leukemic bone marrow cells. In this article we focus on the effects of this treatment on mesenchymal stem cells (MSCs) and monocytes/macrophages; both these cell types are parts of the haematopoietic stem cell niches in the bone marrow. MSCs have unique membrane molecule and constitutive cytokine release profiles, and mediate their support through bidirectional crosstalk involving both cell-cell contact and the local cytokine network. It is not known how various forms of PI3K-Akt-mTOR targeting alter the molecular mechanisms of this crosstalk. The effect on monocytes/macrophages is also difficult to predict and depends on the targeted molecule. Thus, further development of PI3K-Akt-mTOR targeting into a clinical strategy requires detailed molecular studies in well-characterized experimental models combined with careful clinical studies, to identify patient subsets that are likely to respond to this treatment.

## 1. Introduction

The intracellular signalling mediators phosphoinositide 3-kinase (PI3K), Akt (protein kinase B/PKB) and mammalian target of rapamycin (mTOR) form a signalling network rather than a signalling pathway, and, as will be discussed later, targeted therapy directed against members of this network is now considered as a possible strategy in the treatment of human acute myeloid leukaemia (AML). However, this network is not only important in the leukemic cells but also for various non-leukemic cells in the bone marrow (BM). Thus, PI3K-Akt-mTOR targeting will not only affect leukemic cells but also their neighbouring leukaemia-supporting stromal cells [1,2]. In the present review we therefore discuss the role of the PI3K-Akt-mTOR pathway and the complexity of targeting this network in AML; we focus especially on the leukaemia-supporting mesenchymal stem cells (MSCs) that are regarded as important parts of the stem cell niches in the BM but we also describe effects of this therapeutic strategy on the AML cells as well the effects on monocytes because these immunocompetent cells also contribute to the formation of stem cell niches [3].

## 2. PI3K-Akt-mTOR Signalling

**Figure 1:**
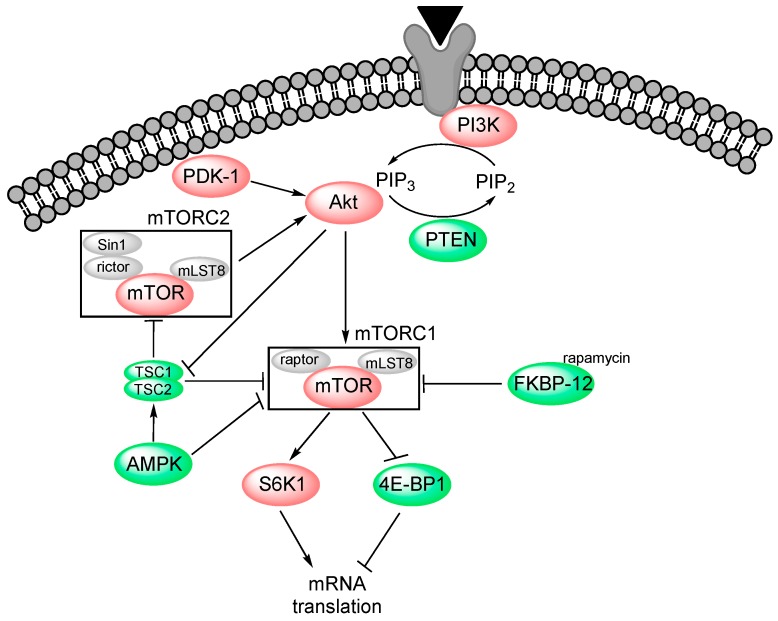
The PI3K-Akt-mTOR pathway. Signalling through this pathway can be initiated through growth factor ligation of specific receptors. PI3K is then activated which leads to formation of PIP3 that activates/phosphorylates Akt. PTEN has PI3K-opposing effect on the equilibrium between PIP2 and PIP3, thereby inhibiting activation of Akt and its downstream partners. Final activation of Akt is mediated by PDK-1 and mTORC2. mTORC1 is activated by Akt, and is inhibited by rapamycin, AMPK and the TSC1/TSC2 complex. Akt is a negative regulator of the latter. Green colour indicates inhibitory activity, red colour indicates activation of signalling through the pathway.

PI3K-Akt-mTOR signalling regulates many key functions in a wide range of cells. The members of this pathway control the expression of proteins that regulate both apoptosis and cell cycle progression/proliferation [4,5], they are important for cell trafficking/mobility and thereby become important for angiogenesis [6], they are important regulators of cellular metabolism [7], and the furthest downstream members of the pathway control protein synthesis and thereby cellular differentiation [5]. An overview of the pathway and how dysregulation of it is involved in many human cancers is given below (see also Figure 1), and the pharmacological targeting of various members/regulators of the pathway is discussed in Section 2.4.

### 2.1. PI3K

The recruitment of PI3K to the plasma membrane is stimulated by growth factors as well as several other cytokines and attachment of the cells to the extracellular matrix [8]. PI3K is activated through auto-phosphorylation [4]. The most important substrate of the kinase is phosphatidylinositol 4,5-bisphosphate (PtdIns(4,5)P_2_ or simply PIP_2_); this mediator is further phosphorylated to phosphatidylinositol 3,4,5-trisphosphate (PtdIns(3,4,5)P_3_ or PIP_3_) [7] which activates Akt and thereby regulates cell cycle progression, apoptosis and the cellular response to insulin [8]. The phosphatase and tensin homolog (PTEN) catalyses the reverse reaction, i.e., dephosphorylation of PIP_3_ back to PIP_2_ [9]. PIP_3_ provides an anchor for several proteins, including Akt and 3-phosphoinositide-dependent protein kinase 1 (PDK-1) domain [7].

### 2.2. Akt (Protein Kinase B)

Akt is one of the key molecules downstream to PI3K. Upon binding to PI3K through its N-terminal pleckstrin homology domain [8], Akt gets activated by phosphorylation at the two sites T308 and S473 that can be phosphorylated independently of each other [10]. Single phosphorylation results in lower activity of Akt [11], but S473 phosphorylation seems to have more influence on substrate specificity than absolute protein activity [12,13]. T308 is phosphorylated by PDK-1, whereas the mammalian target of rapamycin complex 2 (mTORC2) carries out the phosphorylation of S473 [7]. Once activated, Akt translocates from the membrane to the cytosol and the nucleus where it phosphorylates its targets at specific serine and threonine residues [4]. Akt has a substrate specificity towards the motif RxRxxS/Tx_bulk_, where the amino acids at positions four and five preferentially are small and x_bulk_ is an aromatic amino acid [14]. Approximately 9500 human proteins contain such a motif, but Akt also has a protein docking site so that potential substrates not necessarily have to contain this motif [8]. Akt regulates cellular metabolism, growth and survival of many different cell types, including endothelial cells (ECs) responsible for angiogenesis [7]. Induction of such functional effects probably involves modulation of a wide range of proteins, including inhibition of cell cycle arrest by phosphorylating p21 and increased translation rate of cell cycle regulator cyclin D [8].

### 2.3. mTOR

One important substrate of Akt is mTOR, a large protein with a C-terminal serine/threonine PI3K-related kinase domain [2,9,15]. mTOR is a part of the two protein complexes mTORC1 and mTORC2. The former is activated by Akt and acts also as inhibitor of Akt through negative feedback [7], whereas the latter complex phosphorylates Akt at S473 to alter its substrate specificity [15]. The activation of mTORC1 is controlled by the tumour suppressor tuberous sclerosis complexes 1 and 2 (forming the TSC1/TSC2 dimer) that promote the formation of the mTOR complex only upon inhibition by Akt [16].

Binding of rapamycin to FKBP-12 inhibits the activity of the mTORC1 complex [9] that consists of mTOR, regulatory-associated protein of mTOR (raptor) and mammalian lethal with SEC13 protein 8 (mLST8, also called Gβ-like protein/GβL) [13]. Raptor and mLST8 are positive regulators of mTORC1; the former protein binds to S6 kinase 1 (S6K1) and 4E-binding protein 1 (4E-BP1) that are the two most important substrates of the complex, whereas mLST8 stabilizes the association between mTOR and raptor and activates mTOR in the presence of nutrients [9]. On the other hand, the mTORC2 complex is not inhibited by rapamycin [7], and contains mTOR, rapamycin-insensitive companion of mTOR (rictor), mLST8 and stress-activated protein kinase-interacting protein 1 (Sin1) [13]. Rictor, mLST8 and Sin1 are all essential for maintenance and function of the complex [12,17], and Sin1 seems to be the key regulator of its nutrient-independent activation of Akt [13].

mTOR is regulated by insulin and insulin growth factors (IGFs) [13], nutrients (especially amino acids) [18], available ATP [9] and various forms of stress (e.g., hypoxia and DNA damage) [19]. The main task of mTORC1 is to mediate protein synthesis via activation of S6K1 that enhances mRNA translation, and to inhibit 4E-BP1, thus facilitating mRNA binding to the small ribosomal subunit [4]. mTORC1 is also important for ribosome biogenesis and recruitment [9], degradation of cytoplasmic contents upon nutrient starvation (so-called macroautophagy) and the control of trafficking, uptake and metabolism of nutrients such as glucose, amino acids, lipoproteins and iron [18,20]. The signal transducer and activator of transcription 3 (Stat3) is also a substrate of mTORC1 [21]. The function of mTORC2 is activation of Akt and in addition organization of the actin cytoskeleton [15] and regulation of cell survival and metabolism [22].

### 2.4. Pharmacological Targeting of the PI3K-Akt-mTOR Pathway

It can be seen from Figure 1 that the PI3K-Akt-mTOR pathway is part of a signalling network, and the activity is modified by several regulators that target different components of the main signalling pathway. As exemplified in Table 1 [23,24,25,26,27], several different inhibitors have been developed. Some of these mediators inhibit the signalling through inhibition of single signalling mediators in the pathway (i.e., PI3K, Akt or mTOR), whereas others have indirect effects through activation of inhibitory pathway regulators (i.e., PTEN of AMPK). Several combined inhibitors have also been developed. One would expect these diverse mediators to differ in their final effects on pathway signalling and the balance between activation of the various downstream mediators.

The mTOR inhibitors differ with regard to their specificity (i.e., inhibition of mTORC1 alone or dual mTOR inhibition). Additional references together with a more detailed discussion with regard to characterization of the specificity for new mTOR inhibitors are given in previous articles [23,26].

To conclude, targeting of the PI3K-Akt-mTOR pathway is now regarded as a possible therapeutic strategy in many human cancers [28]. Several agents targeting members of this pathway or their regulators have been developed, and as described in detail by Bertacchini et al. [24] many of them are now in clinical trials. Several of these new agents are tried in AML therapy (e.g., AZD-53, CAL-101, MK2206, NVP-BEZ235, OSI-027, PP242).

## 3. Direct Effects of PI3K-Akt-mTOR Inhibition on AML Cells: Patient Heterogeneity and Resistance to Treatment

### 3.1. Dysregulation of PI3K-Akt-mTOR in Human Malignancies—General Comments

Dysregulation of the PI3K-Akt-mTOR pathway, resulting in growth enhancement, resistance to apoptosis and altered metabolism [29], plays a crucial role in the development of various cancers and mutations of proteins belonging to this pathway are, second to mutations in p53, the most frequent alterations in human malignancies [30]. Akt is a central node in tumourigenesis and although the protein itself is rarely mutated [10], it is hyperactivated in many cancers [5] leading to increased cell metabolism, proliferation and survival [9]. Akt overactivation has also been correlated with poor prognosis [5], drug resistance [7] and tumour angiogenesis [8]. Constitutive Akt overexpression/activation is in most cases either a result of a mutation in a tumour suppressor (e.g., PTEN) [5,31] or a potential oncogene (e.g., the p110α regulatory domain of PI3K) [32]. However, single drug treatment of cancers with PI3K-Akt-mTOR dysregulation has proven to be difficult; treatment with rapamycin may knock out mTORC1 completely, but it might also cause enhanced growth factor-initiated activation of PI3K and Akt (e.g., by IGF-1) leading to signalling through other downstream targets than mTORC1, for instance members of the forkhead box (FOX) transcription factor family [29]. Furthermore, the PI3K-Akt-mTOR pathway is intertwined with the mitogen-activated protein kinases-extracellular signal-regulated kinases (MAPK-ERK) pathway, and inhibition of Akt or mTORC1 may therefore lead to MAPK activation and thereby drug resistance [33,34,35]. However, combining PI3K and mTORC1 inhibition may have synergistic effects [29]; the same seems to be true for combined blockage of PI3K-Akt-mTOR and MAPK-ERK [35].

### 3.2. PI3K-Akt-mTOR Targeting in Human AML

As described and discussed in detail in several recent excellent reviews, inhibition of PI3K-Akt-mTOR is now considered as a therapeutic strategy in AML [1,23,25,36,37,38,39]. Several of the aspects for cancer in general (described above) are also relevant for AML. The previous studies of PI3K-Akt-mTOR inhibition as a therapeutic approach in AML have shown the following results:

- 50%–80% of AML patients display Akt that is phosphorylated at T308, S473 or both. This upregulation has been detected not only in bulk AML cells but also in the more immature leukemic stem cells [36,37]. Several studies suggest that both overall and disease-free survival is shorter in patients with PI3K-Akt-mTOR pathway upregulation [36]. In contrast, constitutive activation of the upstream PI3K may represent a favourable prognostic parameter [40].
- The causes for activation of PI3K-Akt-mTOR signalling can be mutations in the FMS-like tyrosine kinase 3 (Flt3), proto-oncogene c-Kit (CD117) or K-Ras genes, overexpression of PI3K or PDK-1, low levels of protein phosphatase 2 (PP2A), autocrine or paracrine release of growth factors (e.g., IGF-1, platelet-derived growth factor/PDGF or the chemokine CXCL12), stromal/fibronectin-induced upregulation of integrin-linked kinase 1 (ILK1), or PTEN loss [36,37]. Activating mutations in PI3K or Akt, however, are uncommon also in AML [36].
- Patients are heterogeneous with regard to the effect of PI3K-Akt-mTOR inhibitors on AML cell proliferation; although an antiproliferative effect is observed for most patients, no effect or even growth enhancement is seen for a subset of patients [27]. This adverse effect is possibly associated with differences in cell cycle regulation.
- There seems to exist several escape mechanisms to inhibition of this pathway [1]. Firstly, induction of autophagy during treatment may represent a mechanism of resistance, and combination of PI3K-Akt-mTOR and autophagy inhibitors has therefore been suggested. Secondly, paradoxical Akt phosphorylation during treatment may induce expression and autophosphorylation of the receptors for insulin, IGF-1 and PDGF resulting in increased pathway activation. This feedback effect can be blocked by PDGFR/IGF-1R/Flt3 inhibition. Thirdly, activation of MAPK-interacting kinases can increase eukaryotic translation initiation factor E4 (eIF4E) phosphorylation and thereby trigger synthesis of pro-survival proteins. Finally, increased signalling of alternative pathways (e.g., ERK upregulation) can also be seen. These observations clearly illustrate the intracellular complexity of PI3K-Akt-mTOR inhibition.
- New mTOR inhibitors seem to target both TORC1 and TORC2, whereas the earlier inhibitors targeted mainly TORC1; the more recent inhibitors may thereby have a stronger effect [23].
- 5′ AMP-activated protein kinase (AMPK) is an inhibitor of mTORC1; directly through inhibition of raptor and indirectly through activation of the TSC1/TSC2 complex [41]. At starvation, AMPK initiates increased fatty acid oxidation and also autophagy, and AMPK activation/agonists have a cytotoxic effect in AML cells [25].
- The combination of conventional chemotherapy with PI3K-Akt-mTOR inhibitors seems to have an acceptable toxicity, but further clinical studies are needed to clarify whether there are additive or synergistic antileukemic effects [38].

Thus, PI3K-Akt-mTOR inhibition is a possible therapeutic strategy also in AML, but with AML being a highly heterogeneous disease the effects may differ among subsets of patients.

## 4. PI3K-Akt-mTOR in MSCs

### 4.1. Identification, Differentiation and Function of Bone Marrow MSCs

MSCs are pluripotent cells capable of self-renewal and differentiation into various cells of the mesenchymal lineages. They were originally identified in vitro as plastic-adherent cells which are able to differentiate into osteoblasts [42] and to form fibroblast colonies (CFU-F) [43]. MSCs are large cells with prominent nuclei [44] and a fibroblast-like morphology [45,46] with mostly spindle-shaped cells [47], but subpopulations of large flat or small round cells can often be detected [48]. Traditionally, MSCs have been linked with the bone marrow (BM MSCs), but they can be isolated from almost every type of connective tissue [47,49], e.g., adipose tissue, dental pulp, synovia, umbilical cord, skeletal muscle in addition to peripheral blood [50,51,52,53,54]. MSCs make up between 0.001% and 0.01% of BM mononuclear cells [50], which is even less than haematopoietic stem cells (HSC) that make up approximately 1% of the BM cells [44].

MSCs are progenitors of cells of the mesenchymal lineages, i.e., osteoblasts, adipocytes, chondrocytes [55], and also fibroblasts and pericytes [50]. However, MSCs have the capacity to trans-differentiate [56], i.e., they can differentiate into lineages outside the mesenchymal remit [2]. So far, MSCs have been shown to trans-differentiate into various neural cells (neurons, astrocytes and glial cells) [57,58], cardiomyocytes [59], skeletal [60] and smooth muscle cells [61], hepatocytes [48], epithelial cells [56] and pancreatic cells [54]. MSCs make up a heterogeneous population with respect to self-renewal and differentiation potential. Thus, not all MSCs fulfil the criteria of stem cells; i.e., some cells show a higher lineage commitment and thereby a reduced self-renewal capability, whereas other cells are more immature and resemble embryonic stem cells, so-called ESC-like cells [52]. MSCs also show reversible commitment, i.e., they can undergo re-differentiation into another lineage in vitro [62,63].

The most important function of BM MSCs is to provide the environment for growth, maturation, differentiation and survival of both normal and leukemic haematopoietic cells [56]. MSCs are also recruited to sites of inflammation where they engraft and repair damaged tissue [2,50], they can act as phagocytes and antigen-presenting cells (APCs) under immunostimulatory conditions [53,64], and they can be recruited to tumours and support cancer cell growth and survival [2,65].

Previous studies have used MSCs isolated from different tissues and with varying potential of self-renewal and differentiation; this is probably the reason why it has been difficult to reproduce the results from many previous studies. The International Society for Cellular Therapy (ISCT) has therefore defined three criteria that MSCs must fulfil: plastic adherence under standard culture conditions, expression of several distinct clusters of differentiation (CDs) and the absence of several haematopoietic markers (see Section 6); and finally, the ability to differentiate into bone, fat and cartilage in vitro [66]. This review focuses on the surface markers of MSCs, their communication with neighbouring cells through constitutive cytokine release and finally, the importance of PI3K-Akt-mTOR signalling for MSC proliferation, differentiation and communication with neighbouring BM cells, including AML cells.

### 4.2. MSC Contributions to Stem Cell Niches in the Bone Marrow

**Figure 2:**
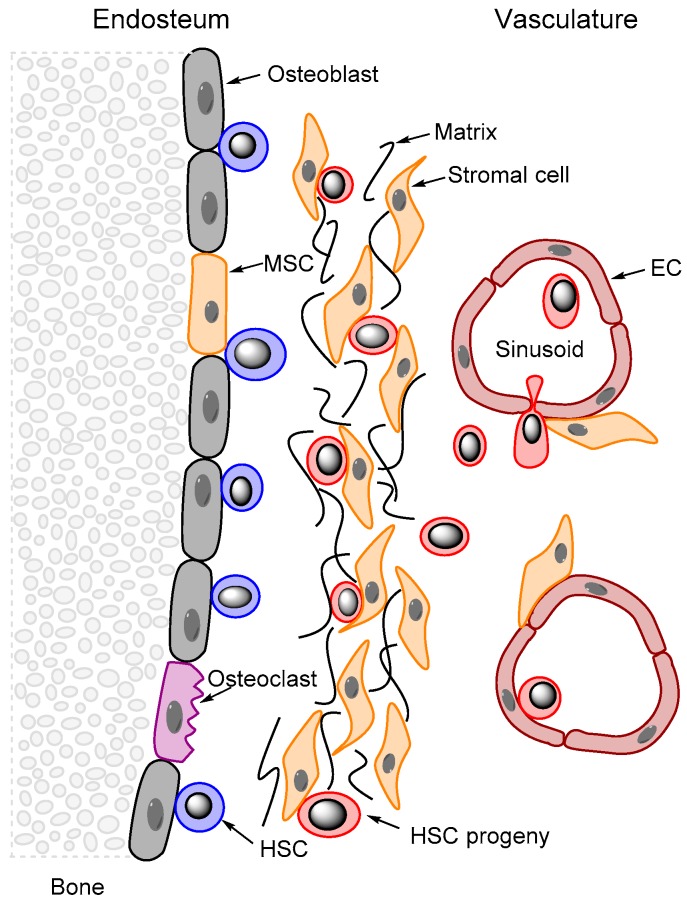
The contribution of MSCs to the microenvironment of bone marrow stem cell niches. Several cell types contribute to the stem cell niches, especially the osteoblasts, MSCs and ECs. The MSCs are localized both close to the sinusoids where they seem to contribute to the regulation of cell trafficking across the vessel wall, and the extravascular space between the vessels and close to the osteoblasts in the endosteum where they also release extracellular matrix molecules. Thus, MSCs are important both for the extravascular microenvironment of the HSCs and for cell trafficking to and from this microenvironment. Monocytes/macrophages and sympathetic nerve fibres are also important for the supportive functions of the stem cell niches; these components are not shown in the figure.

BM MSCs are located mainly within the vasculature, on the surface of the main sinusoidal blood vessels [64], and on the trabeculae [56] where they provide the environment for regulation of haematopoietic stem cell proliferation and quiescence [48,62]. The haematopoietic microenvironment is established by mesenchymal stromal elements which form a complex network of cells, extracellular matrix glycoproteins and growth factors released by the stromal cells [67]; the latter consists of ECs, adipocytes, macrophages, reticular cells, fibroblasts, osteoprogenitors and haematopoietic stem cells with their progeny [62]. The microenvironments, also called niches, embed haematopoietic stem cells and more mature blood cells and protect these cells from differentiation and apoptotic stimuli, thus providing a surrounding for stem cell maintenance and self-renewal [68]. These stem cell niches can be defined as the cellular and molecular microenvironments that regulate stem cell function together with stem cell autonomous mechanisms including the control of the balance between quiescence, self-renewal and differentiation, and responsiveness to environmental stress. The marrow niche is formed by perivascular MSCs together with macrophages, sinusoidal ECs, sympathetic nerve fibres, and cells of the osteoblastic lineage (Figure 2) [3]. The MSCs thereby contribute to the control of stem cell differentiation and the subsequent release of their mature progenies [47].

The best characterized marrow niches are the endosteal and vascular niches [62]. MSC-derived osteoblasts, especially the more immature osteolineage cells [69], are the crucial elements of the endosteal niche through the regulation of the stages of haematopoietic development [53] and the control of the quiescent (G_0_) stage [47]. Additionally, the amount of haematopoietic stem cells within the marrow is affected by changes in the local osteoblast number and function [70]. The vascular or endothelial niche contains discrete areas of endothelium which circulating hematopoietic stem cells preferentially home and engraft to [70]. Thus, in this microenvironment stem cell maintenance [69] and recruitment as well as proliferation and differentiation is regulated [47]. Osteoblasts control the myeloid differentiation of primitive stem cells and the differentiation of B-cells through excretion of granulocyte-colony stimulating factor (G-CSF). On the other hand, adipocytes are negative regulators of stem cell content and function in the marrow [71]. Experimental studies suggest that both osteoblast, fibroblasts and microvascular ECs can support leukemic haematopoiesis (i.e., AML cell proliferation) [72,73,74,75,76], and the vascular niche is also a supportive environment for tumour cells [70] and thereby a frequent site for tumour spread [77]. Finally, MSCs are located close to the sinusoid and are therefore supposed to have a gate-keeper function, i.e., cells entering and leaving the marrow have to by-pass and/or engage with MSCs [64,78]. Thus, MSCs might be actively involved in BM homeostasis as a first line of the immune response [78], e.g., increased release of HSCs into circulation during infections [53]. Cell trafficking leading to release of normal or leukemic cells to peripheral blood as well as homing of circulating tumour cells to the BM also involve MSCs. However, in spite of the well-established view of niches, the true identity of the MSCs and contribution to the niches in vivo is still under debate [52]. For example, MSCs close to blood vessels resemble pericytes in appearance but possess the unique properties and functions of MSCs [45].

## 5. The AML-Supporting Effects of Mesenchymal Stem Cells: Contributions from Cell-Cell Contact and Distant Effects Mediated through the Local Cytokine Network

**Figure 3:**
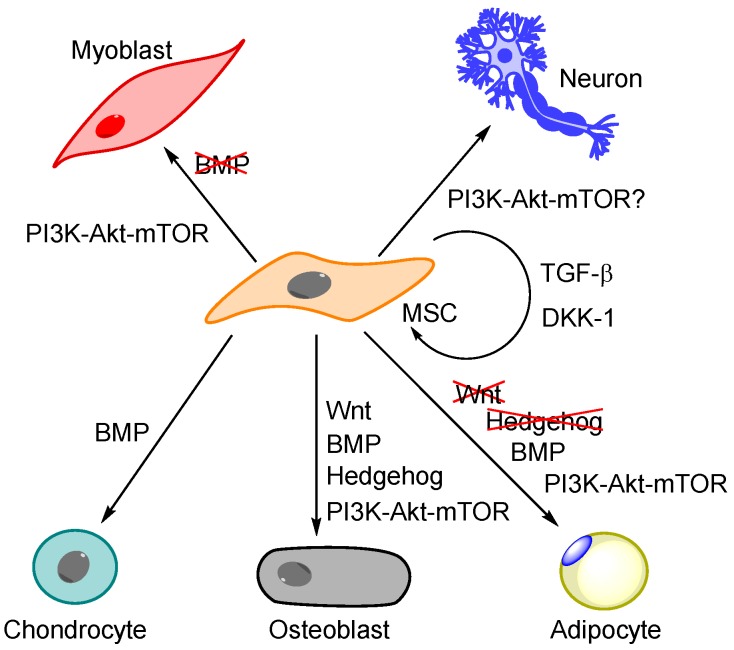
Mesenchymal stem cell self-renewal and differentiation. Transforming growth factor β (TGF-β) and Wingless (Wnt) inhibitor Dickkopf-1 (DKK-1) contribute to MSC self-renewal; whereas signalling through PI3K-Akt-mTOR, Wnt, Hedgehog and bone morphogenetic protein (BMP) is involved in MSC differentiation into the three mesenchymal lineages and furthermore trans-differentiation into myoblasts and neurons. Pathways that inhibit the differentiation into a specific lineage are marked with crosses (e.g., Wnt prevents adipogenesis).

Several signalling pathways are involved in MSC differentiation together with PI3K-Akt-mTOR, including Bone Morphogenetic Protein (BMP) signalling, the Wingless (Wnt) pathway and Hedgehog signalling (Figure 3). Most pathways exhibit an inverse relationship on differentiation, i.e., they support differentiation into one lineage and suppress other lineages [79].

### 5.1. Regulation of MSC Differentiation Is More Than PI3K-Akt-mTOR—The Importance of BMP, Wnt and Hedgehog Signalling

BMPs are multifunctional cytokines that belong to the transforming growth factor β (TGF-β) superfamily, and this family together with the Wnt pathway are crucial for MSC development and maintenance [80]. TGF-β and Wnt cooperatively stimulate MSC self-renewal and inhibit differentiation into osteoblasts and adipocytes [81]. In concurrence with this, inhibition of TGF-β leads to osteoblast [82,83] and adipocyte differentiation with suppressed chondrogenesis [84]. On the other hand, BMP-2 promotes osteogenesis [85] and enhances osteoblast function [86]. The lineage commitment also depends on cell density: at a low cell concentration, the BMP pathway leads to adipogenesis, whereas a high cell density favours differentiation towards osteoblasts and chondrocytes [55,87]. Interestingly, both MSCs and osteoblasts themselves produce proteins of the TGF-β superfamily [79], and MSCs also express at least four Wnt members [82]. The Wnt pathway as well as the Hedgehog pathway stimulate osteogenesis while suppressing adipogenesis [79], and the Wnt inhibitor Dickkopf-1 enables MSCs to enter the cell cycle, thus inhibiting osteoblast differentiation [88]. The Wnt3a protein promotes MSC self-renewal by increasing proliferation while simultaneously decreasing apoptosis and osteogenesis [62].

### 5.2. The General Effect of PI3K-Akt-mTOR Signalling on MSC Differentiation

Adipogenic and osteogenic differentiation are controlled by IGF-1R via its direct or indirect, activation of PI3K, PDK-1, Akt and mTOR [79]. A critical level of Akt activity is required for adipogenesis [79], and PI3K, Akt, mTORC1 and S6K1 are all activated during adipogenesis [89]. The PI3K-Akt-mTOR pathway is also a positive regulator of terminal adipocyte differentiation [89], whereas S6K1 is important for commitment to the adipogenic lineage [90]. Concurrent with these observations, both rapamycin [2,89,91] and the loss of raptor [22] reduce adipogenesis, whereas the loss of rictor promotes adipogenesis on behalf of osteogenesis [22]. Additionally, mTORC1 is required for osteoblast proliferation and differentiation, and also regulates osteoblastic function [2]. The PI3K-Akt-mTOR and the BMP pathways (see below) interact; BMP-2 activates Akt to induce osteoblast differentiation and also activates PI3K in osteoblasts, whereas PI3K and Akt are required for the expression of the BMP-2 osteoblast differentiation marker and are also necessary for the transcription of BMP-2 [86]. Finally, regarding differentiation into unrelated germline lineages, both mTOR complexes as well as PI3K seem to be involved in myogenesis [2,86], whereas BMPs suppress this differentiation [83]. Other lineage differentiations, like those into neural, pancreatic and endothelial cells seem to be effects of silenced or forced expression of single transcription factors [54], but differentiation into neurons has also been correlated with mTOR [2].

### 5.3. The Unique Membrane Molecule Profile of MSCs: Possible Molecular Mechanisms for Communication with Neighbouring Cells through Direct Cell-Cell Contact and via the Local Cytokine Network

MSCs express a variety of surface markers and receptors but no single marker is MSC-specific (Table 2) [50,52,53,80,92,93]. These markers include several molecules involved in cell-cell and cell-matrix contact, receptors for soluble mediators and various enzymes that can modulate intercellular communication. According to the ICST criteria [66] a MSC cell population has to show at least 95% of CD73^+^ (ecto 5′ nucleotidase), CD90^+^ (Thy-1) and CD105^+^ (endoglin) cells, whereas less than 2% of cells may express the pan-leukocyte marker CD45, the immature haematopoietic cell marker CD34, the monocyte/macrophage markers CD11b and/or CD14, the B-cell markers CD19 and/or CD79, and major histocompatibility complex (MHC) class II HLA-DR molecules; the latter are only expressed after MSC stimulation with for example interferon γ (IFN-γ) [66]. MSCs have furthermore been proposed to be negative for several other markers commonly expressed on other lineages (summarized in Table 1): endothelial progenitor marker CD31 (PECAM-1) [94], myeloid marker CD15 (SSEA-1) [95], haematopoietic marker CD133 [96], T-cell markers CD3, CD4 and CD8, natural killer (NK) cell marker CD56 (NCAM) [95], B-cell markers CD80 [47], antigen-presenting cell markers CD40 and CD86 [93], erythrocyte marker CD235a [80], and CD117 [94]. 

However, among these markers CD31 [55], CD56 [48] and CD117 [99] have also been proposed as potential MSC markers, with CD56 possibly being a stemness-maintaining factor [48]. Furthermore CD105 is a marker that has to be present on MSCs, but it is also expressed by ECs and pre-mature B-cells [96]. These observations further illustrate that combinations of markers have to be used to identify MSCs. There is also a long list of membrane molecules that can be expressed by MSC or subsets of MSCs (recently reviewed in [52]), including: CD10 (neprilysin), CD13 (alanine aminopeptidase) [96], CD71 (transferrin receptor protein 1) [47], cytokine receptor CD135 (Flt3) [55], BM stromal cell antigen CD157 [95], CD271 (LNGFR) [96], CD349 (frizzled-9) [52], HCAM (CD44) [50], VCAM-1 (CD106), MCAM (CD146) [98], ALCAM (CD166) [46], ICAM [97], STRO-1 [67], the neural ganglioside GD2 [48], pericyte marker 3G5, SSEA-3 [52], SSEA-4 [80], cadherin-11, N-cadherin (CD325) [48], L- and P-selectin (CD62L/P) [55], thrombospondin-1 [48], and integrins α1, α5, α6 and β1 (CD49a/e/f and CD29, respectively) [48,52,95]. MSCs also express some intracellular markers like nestin [52], sex-determining region Y-box 2 (Sox-2) and octamer-binding transcription factor 4 (OCT-4) [50], and a variety of chemokine receptors [93,94,95,100].

There is no general agreement about which of the markers that are best in determining stemness. Some authors advise not to use universally expressed markers that are not unique for this cell type; this includes 3G5, GD-2, CD349, CD271, STRO-1 and SSEA-4, even though the last three are important for MSC clonogenecity and function [52], and SSEA-4 can be useful for separating MSCs from HSCs [80]. On the other hand, the cadherins, integrins and thrombospondin have potential as stemness markers [48] and are associated with clonogenecity and multipotency [52]. The presence of VCAM-1 indicates that cells are multipotent and support haematopoiesis; it is also expressed by most MSCs, and might thus be the most suitable stemness marker [48,52]. The most immature MSCs express several markers that are lost upon commitment and may thus be used to identify a subset of pluripotent MSCs; these markers are SSEA-3 [52], Sox-2, OCT-4 [50] and, probably, nestin [52].

MSCs do not only lose primitive stemness markers upon differentiation but also upon passaging in vitro [80,95,96], including the chemokine receptor CXCR4 which is important for cell migration [45,97]. Several markers are also lost when cells reach confluence [52]; and at the same time the cells may lose their differentiation potential [48]. However, certain markers can also be gained, both in vitro [80,95,96] and in the BM niches [44].

MSC homing involves several molecular mechanisms [45,99], including various chemokines [47] and adhesion molecules [80]. The most important chemokine, which is expressed at low concentrations by MSCs themselves, seems to be CXCL12; this CXCR4-ligand induces MSC homing, migration and marrow engraftment [99,101] in a dose-dependent manner [102]. MSCs adhere to ECs [80,98], and both integrins and the adhesion molecules P-selectin and VCAM-1 are important for their adhesion to endothelium [93]. However, MSCs do not only migrate to the BM [94], they also have multi-organ homing capacity [68] and can migrate to lymphoid organs through CCR7 and CXCR5 ligation, to the skin through CCR4 and CCR10 ligation, and to the small intestine and salivary glands through CCR10 ligation [100]. They also show chemokine-mediated migration to sites of wound healing and tissue regeneration [78] where they provide a microenvironment favouring tissue regeneration [103].

### 5.4. The Functional Importance of PI3K-Akt-mTOR Signalling in MSCs: The Effects on MSC Differentiation Are Only a Part of a More Extensive and Complex Biological Impact

The PI3K-Akt-mTOR pathway is important for the regulation of several biological characteristics of the MSCs; the current knowledge is described more in detail in Table 3 [2,76,91,104,105,106,107,108,109,110,111,112,113,114,115,116,117,118,119,120,121,122,123,124,125,126,127,128,129,130,131,132,133,134]. Firstly, the pathway is important for stress responses, including the adaptation of the MSCs to the hypoxic BM microenvironment. It is also important for regulation of MSC metabolism and induction of autophagy. Secondly, MSCs show functional differences depending on the patients’ age, and altered activation of the pathway is involved in the process of aging and age-dependent differences of BM MSCs. Thirdly, the various parts of this pathway are also involved in the process of MSC differentiation. The most important differentiated mesenchymal cells in the BM are AML supporting osteoblasts and adipocytes [72], and the PI3K-Akt-mTOR network is important for the balance between osteogenic and adipogenic differentiation. Finally, the pathway contributes to the regulation of MSC communication between MSCs and their neighbouring cells, including more differentiated AML-supporting cells (e.g., osteoblasts and fibroblasts).

Thus, PI3K-Akt-mTOR signalling is important both for aging, adaptation, differentiation, communication and proliferation of BM MSCs, and even though the molecular mechanisms are only partly known, one would expect PI3K-Akt-mTOR inhibition to alter the MSC functional characteristics and thereby also affect the AML cell microenvironment including that of the stem cell niches. There is a molecular crosstalk between AML cells and MSCs, and studies of the global gene expression profiles suggest that for most patients this crosstalk results in an altered local cytokine network mediated through effects on NFκB signalling but without induction of osteoblastic or adipogenic differentiation [135]. It is not known how PI3K-Akt-mTOR inhibition will alter this crosstalk. Finally, one would also expect differences among patients because even though previous studies of gene expression suggest that the effects of this crosstalk on the MSCs are similar for most patients, there are exceptional patients showing distinct effects of the AML-MSC crosstalk on gene expression [135].

### 5.5. Cytokine-Mediated Communication between MSCs and the Neighbouring Bone Marrow Cells; a Part of the AML-Supporting Effects by the MSCs

MSCs release a wide range of cytokines (Table 4) that are important for communication with neighbouring cells [47] or are parts of autocrine loops [48]. The cytokines are important for the support of haematopoiesis [136,137], for initiating tissue regeneration [45,138] and for immunoregulation [51]. This constitutive release is a part of a bidirectional cytokine-mediated crosstalk between MSCs and AML cells, and its final effect is to support the growth and survival of human AML cells, including the long-term proliferating leukemic stem cells. This supporting effect is detected for most patients, but there is also a minority of patients where this effect is not observed (Brenner, Revised Version submitted).

## 6. Direct Effects of PI3K-Akt-mTOR Inhibition on Immunocompetent Cells and the Dual Function of Monocytes/Macrophages as Immunocompetent Cells and Members of the Stem Cell Niches

**Figure 4:**
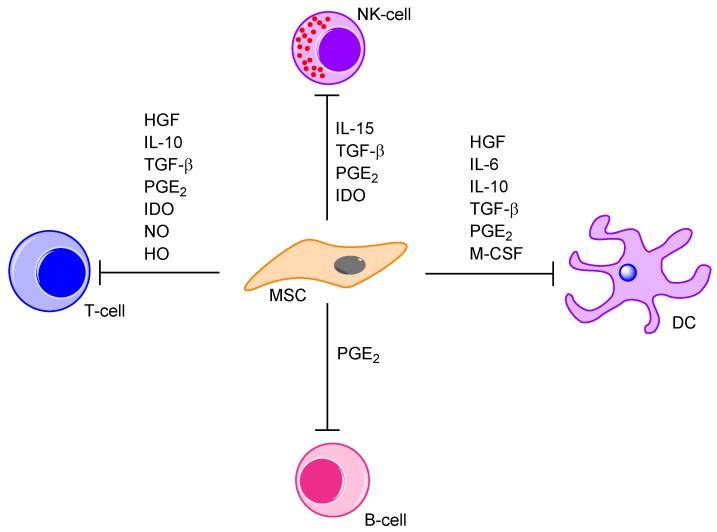
Immunosuppressive effects of mesenchymal stem cells. MSCs excrete various cytokines that mediate immunosuppression of T-cells, B-cells, natural killer cells (NK) and dendritic cells (DC). Some mediators, like prostaglandin E2 (PGE_2_), exhibit an immunosuppressive effect on all immune cells, whereas others, like IL-15, are specific inhibitors of single cell types.

Several soluble mediators derived from MSCs are probably important for their immunoregulatory functions [53,78,101] and the MSC-induced downregulation of various immunocompetent cells [53]. The most important effects on T-cells, B-cells, monocytes/macrophages and dendritic cells are summarized in Table 5 and Figure 4 [45,47,51,53,68,71,78,80,88,93,136,137,139,140,141,142,145,146,147,148,149,150,151,152]. The effects on T cells are best characterized and some of the other effects may be indirectly caused by T cell inhibition. The BM niche provides a supporting environment for the development of immature T-cells outside the thymus [153], and MSCs can suppress T-cell proliferation [68,137,154] and activation [53]. The inhibition of naïve and memory T-cells seems to be independent of MHC molecules [101] and may involve a proapoptotic effect [68,154], T-cell anergy [80] and/or altered cell cycle regulation and cell cycle arrest in G_0_ or G_1_ phases [147]. The in vitro immunomodulation may involve both direct cell-cell contact and soluble factors [97]. These effects are mainly characterized in experimental models, and it has been suggested that these effects are less important in vivo due to the low number of MSCs [154]. Modulation of these MSC-associated immunoregulatory mechanisms may thereby indirectly alter the function of a wide range of immunocompetent cells.

mTOR inhibitors are now used as immunosuppressive agents in clinical medicine; the rationale for this is their direct effects on a wide range of immunocompetent cells leading to a final effect of immunosuppression. The direct effects of PI3K-Akt-mTOR inhibition on immunocompetent cells have been described in detail in recent reviews [155,156]. In our present article we will have a focus on the monocytes that have a dual function as immunocompetent cells and as a part of the microenvironment in the BM stem cell niches.

### 6.1. PI3K-Akt-mTOR, MSCs and Immunocompetent Cells; Regulation of Allogeneic and Autologous Antileukemic Immune Reactivity

Dependent on the activation state of the immune cells and cytokines they were exposed to—in particular IFN-γ and TNF-α [157], MSCs might also contribute to a pro-inflammatory environment [152]. Cancer cells can alter the MSC cytokine expression profile towards a tumour-supporting microenvironment [136,144]; this is similar to the effects on AML cells as described above. The soluble factors influence several signalling pathways. For instance, IL-6 expression activates signalling through Janus kinase 2 (JAK2) and Stat3 [158,159], EGF increases the concentration of pre-oncogenic phosphorylated Akt [143], whereas CXCL12 and CCL5 stimulate tumour growth through the PI3K-Akt-mTOR pathway [144]. CCL5 in particular has been linked with tumour cell proliferation and metastasis [50]. TGF-β is expressed in tumour cells and tumour stroma and enhances tumour invasion and metastasis, and also suppresses NK-cells [141]. However, MSCs may also induce the immune response in order to reduce tumour growth by excretion of a variety of ILs in combination with IFN-γ and G-CSF [160].

In allogeneic stem cell transplants, MSCs both enhance marrow engraftment and reduce the risk of graft-versus-host-disease (GVHD) [84]; however, at the same time, they may increase the risk of relapse due to a reduced graft-versus-leukaemia response [101]. Immune-mediated antileukemic reactivity is best documented for allografted AML patients, but the experience from autotransplanted patients suggests that autologous antileukemic immune reactivity may also be important [161,162,163]. Furthermore, MSCs are used for suppression of autologous immune reactivity, e.g., in the treatment of auto-immune diseases like Crohn’s disease [78], Parkinson’s disease and diabetes [54]. Their differentiation potential makes them useful in repair and replacement of tissues [62], both within and outside the mesenchymal lineages [54,82,92,94,164]. MSCs have also been proposed as delivery vehicles that home to sites of injury or cancer [93,97]. Whether injected MSCs will support or suppress human malignancies is difficult to predict and probably depends on factors like tumour type, the MSC culture, the amount of engrafted cells and the tumour microenvironment [93,97].

### 6.2. The Role of AKT/mTOR as Regulators of Macrophage Metabolism and Cytokine Release

Macrophages and monocytes need to adapt to a wide variety of metabolic changes during migration, cytokine production and differentiation, and the PI3K-Akt-mTOR pathway is a key regulator in these processes. Classically, macrophages can be divided into non-polarized M0 macrophages and the M1 and M2 macrophage phenotypes [165,166,167]. M1 macrophages are associated with acute antimicrobial defence, production of pro-inflammatory cytokines (IL-6, IL-12 and TNF-α) and nitric oxide (NO). M2 macrophages produce ornithine rather than NO and anti-inflammatory cytokines (e.g., IL-10). Toll-like receptor (TLR) activation by stimulation through lipopolysaccharides (LPS) triggers M1 differentiation via the NFκB pathway and interferon regulatory factors (IRFs), but also through direct activation of the mTOR pathway. Stat6 and C/EBPβ activation by IL-4 and IL-13 are the main factors promoting M2 differentiation; both these mediators are directly affected by mTORC1-mTORC2 regulated intracellular networks. Finally, PI3K also influences macrophage functions via Janus kinases, p38 and ERK.

Cytokine production and metabolism are closely linked in macrophages. M1 macrophages are characterized by glycolysis and a broken Krebs cycle with accumulation of citrate and succinate, whereas M2 macrophages use aerobe glycolysis, β-oxidation and increased glutamine metabolism [168]. The accumulation of succinate in M1 macrophages is essential for IL-1β secretion through induction of hypoxia inducible factor 1α (HIF-1α). In addition, lipidogenesis is necessary for adequate phagocytic function, expansion of intracellular organelles and sustained cytokine production through the production of inflammatory lipid mediators (e.g., prostaglandins) [169]. The role of Akt/mTOR in these processes is not known but the following mechanisms have been suggested [156,169]:

- Increased glycolysis in response to TLR stimulation can be mediated by Akt independent of mTORC1.
- 4E-BP1 and 6SK1 have important roles in controlling the synthesis of both cytokines as well as HIF-1α and IRF-7 that are necessary for the cytokine synthesis.
- Activation of sterol regulatory element-binding protein 1 (SRBP1) is important for synthesis of lipid mediators and cytokines, and also for activation of the pentose-phosphate pathway that is required for adequate respiratory burst.
- mTORC1 is critical for control of glutamine metabolism that again is a regulator of the hexosamine pathway and the processes securing sufficient succinate accumulation.

Taken together these observations illustrate that PI3K-Akt-mTOR inhibition is likely to affect the communication between monocytes and their neighbouring cells (e.g., AML cells), but the final effects are difficult to predict and will probably depend on the mediator in the pathway being targeted.

### 6.3. The Role of Akt/mTOR in Macrophage Polarization—The Importance for AML Cells and Immunoregulation

The role of the PI3K-Akt-mTOR pathway in macrophage differentiation has been investigated in several murine knockout models (Table 6). Whether M1 or M2 polarization is induced depends on the molecule to be targeted. Deletion or modulation of Akt through AMPK deletions lead to loss of M2 polarization accompanied by increased production of pro-inflammatory cytokines [170,171]. On the other hand, deletion of PTEN leads to increased M2 polarization [172,173]. In addition, experimental data suggest that different Akt isoforms have different effects with regard to macrophage polarization, i.e., M1 polarization is Akt2-dependent whereas M2 polarization is Akt1-dependent [174,175], and the Akt2 isoform selectively controls the C/EBPβ expression required for M2 polarization [174]. Finally, TSC1/2 deletion downstream to Akt is associated with an inflammatory M1 phenotype [176,177,178], whereas disruption of the mTORC1 complex by knockout of raptor leads to increased Akt activity and increased M2 polarization [179]. These data clearly illustrate that whether PI3K-Akt-mTOR targeting causes M1 or M2 polarization of monocytes depends on which mediators are targeted.

### 6.4. The Effect of Pharmacological Inhibition of Akt/mTOR in Macrophages

Pharmacological inhibition of the mTOR pathway has yielded conflicting results. Most studies with mTOR inhibitors, however, have shown an increased secretion of pro-inflammatory cytokines like TNF-α, IL-6 and CXCL8, reduced secretion of IL-10, inhibition of macrophage migration, failure to polarize in the M2 direction and increased apoptosis of M2 cells (Table 7) [180,181,182,183,184,185,186,187]. These data support the observation from clinical experience that mTOR inhibitors are associated with several pro-inflammatory side effects [155], including increased systemic levels of several pro-inflammatory cytokines even when mTOR inhibition is combined with steroids [188,189].

The in vivo effects of rapamycin on human M1/M2 also favours M1 polarization and induces increased release of M1-type cytokines (CXCL9, CXCL10, IFN-γ, G-CSF and IL-1ra) in response to LPS stimulation and reduced ability to develop in the direction of M2 polarization [185].

## 7. Summarizing Discussion

### 7.1. The Complexity of PI3K-Akt-mTOR Signalling—What Is the Optimal Molecular Target for Pathway Inhibition?

PI3K-Akt-mTOR is not only a pathway but a signalling network including feedback loops that will alter the continued signalling; it will thereby be important which target within the pathway that is selected for inhibition of the signalling. One example of this is the increased activation of Akt that may be seen after mTOR inhibition; an effect which is not seen after PI3K inhibition and these two strategies may thereby differ in the activation of alternative downstream targets to Akt. Despite this, we previously observed that the final effect on AML cell proliferation was very similar for PI3K, Akt or mTOR inhibition in individual patients, even though the effect varied among patients and both antiproliferative effects and growth enhancement could be detected [27]. These observations suggest that despite the complex direct effects of different strategies on intracellular signalling of the leukemic cells, the final effect on AML cell proliferation is less dependent on which mediator is targeted.

As outlined in Section 2.4 and Table 1, many different inhibitors are now being developed, including inhibitors to various single mediators, dual inhibitors and agonists to inhibitory regulators of the pathway. It is difficult to predict the functional effects of each inhibitor in primary human AML cells, and possibly the optimal therapeutic approach/agent will differ among patients. Additional experimental studies as well as careful clinical studies including detailed biological characterization of the treated patients will be essential to answer these questions.

### 7.2. The Complexity and Heterogeneity of Human AML; the Consequences from PI3K-Akt-mTOR Inhibition in the Leukemic Cells with Regard to Communication with Neighbouring Cells May Differ among Patients

AML is a very heterogeneous disease, and, as pointed out above, patients differ with regard to the effect of PI3K-Akt-mTOR inhibition on AML cell proliferation. However, AML cells differ considerably with regard to the expression of various cell surface molecules (adhesion molecules, cytokine receptors) [193,194] as well as the constitutive release profile [195]. Due to these variations between primary AML cells their communication with leukaemia-supporting neighbouring cells in the BM microenvironment will also differ, and the direct effects of PI3K-Akt-mTOR inhibition on the AML cell communications with neighbouring cells will thereby be difficult to predict and may even increase the patient heterogeneity with regard to the final effect of the treatment on the AML cells.

The importance of targeting the neighbouring leukaemia-supporting microenvironment is clearly illustrated by the use of new targeted therapies in chronic lymphocytic leukaemia [196,197]. Both the Bruton tyrosine kinase inhibitor ibrutinib and the PI3K inhibitor idelalisib are now approved for treatment of this disease, and inhibition of the communication between the leukemic and the neighbouring stromal cells seems important for their antileukemic efficiency. As described in our present review and discussed more in detail in a recent article [193] similar mechanisms may be important in AML. Furthermore, oral administration is possible for both drugs and the toxicity is acceptable; they may therefore become useful in low-toxicity AML stabilizing treatment in elderly and unfit patients [197,198].

### 7.3. The Complex Effects of PI3K-Akt-mTOR Signalling in MSCs

The microenvironment of a tumour resembles that of a chronic wound where MSCs are attracted by the local release of inflammatory chemokines [65,199], such as chemokines [93], growth factors [50], and pro-inflammatory IL-6 [93]. The recruited MSCs may then differentiate into tumour-associated fibroblasts and pericytes [50,65] that support the cancer cells indirectly (e.g., induction of angiogenesis) [65] or directly through growth factor release [50,158,200]. Pericytes can produce extracellular matrix components that further support angiogenesis [50,65]. Similar mechanisms may also be operative in human AML [138]. Furthermore, the fraction of mature osteoblasts relative to immature MSCs seems to be substantially higher in AML than normal BM [22] and this may further support [50] and favour the growth of the leukemic cells over normal hematopoietic cells [201]. Finally, it should be pointed out that BM MSCs in AML include cells with abnormal karyotype [202], but the clinical importance of this observation is uncertain because these alterations thus far could not be linked with prognosis. These abnormal cells can support growth and development of malignant cells [65], but normal MSCs can also support the proliferation of primary human AML cells (Brenner, Revised Version submitted).

MSCs express a wide range of surface molecules that may be involved in the crosstalk with neighbouring cells, e.g., AML cells. Even though PI3K-Akt-mTOR is important for the differentiation of MSCs, very little is known about the effect of this pathway on the expression of single surface molecules or single soluble mediators. It is therefore difficult to predict based on the current knowledge how PI3K-Akt-mTOR inhibition will alter the ability of MSCs to communicate and thereby support AML cells in the BM niches through direct cell-cell contact or through their release of soluble mediators.

### 7.4. The Complex Effects of PI3K-Akt-mTOR Signalling in Macrophages and the Role of Other Immunocompetent Cells

PI3K-Akt-mTOR is important for macrophage polarization, and targeting of this pathway may even increase macrophage release of AML-supporting soluble mediators. Thus, targeting of this pathway will alter the ability of these cells to communicate with neighbouring AML cells in the stem cell niches, but due to the patient heterogeneity in AML the consequences of the induced polarization/inhibition may differ among patients.

Our previous experimental studies have demonstrated that coculture of primary human AML cells and BM MSCs increase the levels of several cytokines in their common microenvironment, including the levels of several chemokines that are important for T cell trafficking [135,203]. Even though only the macrophages are important in the formation of the stem cell niches (see above), other immunocompetent cells may also be important for leukemogenesis or chemosensitivity. Recent studies of auto-transplanted patients suggest that the antileukemic reactivity of autologous T cells is important for the effect of intensive chemotherapy in AML [162]. On the other hand, previous studies suggest that T cells may support leukemogenesis or be important for chemosensitivity in human AML because activated T cells can release several cytokines that function as growth factors for primary human AML cells, e.g., G-CSF and IL-3 [204,205,206,207,208]. Thus, it is not known how the various strategies for inhibition of PI3K-Akt-mTOR will influence the balance between antileukemic and potentially AML-supporting activity by immunocompetent BM cells (e.g., monocytes/macrophages, T cells).

## 8. Final Conclusions

Based on the experimental studies reviewed in this article it is likely that the effect of PI3K-Akt-mTOR inhibition will differ among AML patients. Thus, the optimal design of future clinical studies will require additional experimental investigations to characterize in detail the direct and indirect effects of this treatment on the AML cells. Such studies are necessary in order to clarify which patients are likely to benefit from this treatment, and which pathway mediator is to be targeted in order to achieve maximal antileukemic effect for each individual patient.

## Figures and Tables

**Table 1:** Pharmacological targeting of the PI3K-Akt-mTOR—an overview of various pharmacological agents directed against different mediators/regulators of the pathway [23,24,25,26,27].

Direct Inhibition of the PI3K-Akt-mTOR Pathway Members
**PI3K inhibitors**
Pan-PI3K inhibitors: buparlisib, pilaralisib, pictilisib
Isoform-specific inhibitors: alpelisib, tazelisib, CAL-101, GDC-0941
Others: MVP-BAG956 (PI3K-PDK1), resveratrol (PI3K/Akt)
**Dual PI3K-mTOR inhibitors**
NVP-BEZ235, LY3023414, GSK2126458
**Akt inhibitors**
MK-2206, uprosertib, ipatasertib, AZD5363
**mTORC1 inhibitors**
Sirolimus, everolimus, temsirolimus, ridaforolimus
**Dual mTORC1/2 inhibitors**
LNK128, AZD8055, MLN0138, CC-223
**Indirect Inhibition—Activation of Pathway Inhibitors**
AMPK agonists: metformin, A-769662GSK621
PTEN activation: l-sercurinine

**Table 2:** The cell surface molecular profile of human MSCs; an overview of different molecules. The molecules suggested to be used for identification of MSCs are marked in bold.

Adhesion Molecules and Other Cell Surface Molecules Involved in Local Cell-Cell or Cell-Matrix Contact		
**CD90: Thy-1**		A cell surface glycoprotein and member of the immunoglobulin superfamily involved in cell adhesion and cell communication in numerous cell types, including stem cells.
CD29: Integrin β1 [48]		Integrins are heterodimeric proteins that mediate bidirectional communication across the cell membrane. They are made up of α and β subunits. At least 18 α and eight β subunits have been described. This protein is a β subunit.
CD31: PECAM-1 [55]		This is a cell surface protein; it can be a part of intercellular junctions and is probably involved in leukocyte migration and integrin activation.
CD44: HCAM [50]		This cell-surface glycoprotein is a receptor for hyaluronic acid and is involved in cell-cell interactions, cell adhesion and migration. It can also interact with other ligands, such as osteopontin, collagens, and matrix metalloproteinases (MMPs).
CD48a/e/f: Integrins α1/α5/α6 [48,52]		These integrins are members of the immunoglobulin-like receptor family; it does not have a transmembrane domain, however, but is held at the cell surface by a GPI anchor via a C-terminal domain which may be cleaved to yield a soluble form of the receptor.
CD54: ICAM-1 [97]		This cell surface glycoprotein binds to integrins of type CD11a /CD18, or CD11b/CD18.
CD56 NCAM [48]		This cell adhesion protein is a member of the immunoglobulin superfamily, and it is involved in cell-cell as well as cell-matrix interactions.
CD62L/P: L/P-selectin [55]		CD62P: This 140 kDa membrane protein is a calcium-dependent receptor that binds to sialylated forms of carbohydrate antigens. CD62L: This cell surface adhesion molecule can mediate binding of leucocytes.
CD106: VCAM-1 [98]		This member of the Ig superfamily is a cell surface sialoglycoprotein mediating cell-cell adhesion and signal transduction.
CD146: MCAM [98]		Probably acting as a cell adhesion molecule.
Cadherin-11/Cadherin-2 [48]		Cadherin-11 is a type II classical cadherin from the cadherin superfamily, integral membrane proteins that mediate calcium-dependent cell-cell adhesion. Type II (atypical) cadherins are defined based on their lack of a HAV cell adhesion recognition sequence specific to type I cadherins. This cadherin seems to have a specific function in bone development. Cadherin-2 is a classical cadherin, i.e., a calcium-dependent cell adhesion molecule and glycoprotein.
**Cell Surface Cytokine Receptors**		
**CD95: Endoglin RNG**	A homodimeric transmembrane protein, it is a component of the transforming growth factor β (TGF-β) receptor complex	
CD71: Transferrin receptor protein 1 [47]	This cell surface receptor is necessary for cellular iron uptake by the process of receptor-mediated endocytosis.	
CD117: c-Kit [99]	This protein is the receptor for the stem cell factor (SCF).	
CD135: Flt3 [55]	This receptor is activated by binding of the Flt3 ligand.	
CD166: ALCAM [46]	This protein is a member of a subfamily of immunoglobulin receptors and is a CD6 receptor; it is implicated in cell migration.	
CD271: LNGFR [96]	This is the nerve growth factor receptor.	
CD349: Frizzled-9 [52]	Members of the ‘frizzled’ gene family encode transmembrane proteins that are receptors for Wnt signalling proteins.	
CCR1/4/6/7/9/10 CXCR4/5/6 CX3CR1 [94,95,100]	These are all chemokine receptors that can bind a wide range of CCL and CXCL chemokines; mediators which are important regulators of cell trafficking, cell cycle progression and cell survival.	
**Enzymes**		
**CD73: ecto 5′ nucleotidase**	A plasma membrane protein that catalyses the conversion of extracellular nucleotides to membrane-permeable nucleosides.	
CD10: Neprilysin [96]	A glycoprotein that is a neutral endopeptidase that cleaves peptides at the amino side of hydrophobic residues and inactivates several peptide hormones	
CD13: Alanine aminopeptidase [96]	A plasma membrane protein; the large extracellular carboxy-terminal domain contains a pentapeptide consensus sequence characteristic of members of the zinc-binding metalloproteinase superfamily. The enzyme was thought to be involved in the metabolism of regulatory peptides.	
Thrombospondin [48]	This protein has several distinct regions, including a metalloproteinase domain, a disintegrin-like domain, and a thrombospondin type 1 motif.	
**Additional MSC-Expressed Molecules**		
CD157: Stromal cell antigen [95]	This glycosylphosphatidylinositol-anchored molecule can facilitate cell growth.	
Nestin [52]	This protein is a member of the intermediate filament protein family.	
Sox-2 [50]	This protein is a member of the SRY-related HMG-box (SOX) family of transcription factors required for stem-cell maintenance.	
OCT-4 [50]	This transcription factor is important for stem cell pluripotency.	

The following MSC markers have also been described in previous studies: GD-2 [48], STRO-1 [67], 3G5 [52], SSEA-3 [52] and SSEA-4 [80]. Abbreviations: CAM: cell adhesion molecule; PECAM: platelet endothelial CAM; HCAM: haematopoietic CAM; ICAM: intracellular AM; NCAM: neural CAM; VCAM: vascular CAM; MCAM: melanoma CAM; ALCAM: activated leukocyte AM; LNGFR: low-affinity nerve growth factor receptor; SSEA: stage-specific embryonic antigen.

**Table 3:** PI3K-Akt-mTOR signalling in MSCs; the effects on important biological functions.

**Aging of MSCs: PI3K-Akt-mTOR Inhibition Maintains an Immature State**
BM MSCs show decreased self-renewal, differentiation and function with aging; inhibition of the PI3K-Akt-mTOR pathway preserves the immature state and prevents the development of the age-related phenotype [109]. Increased expression of the transcription factors NANOG and OCT-1 may be responsible for this. Furthermore, a comparison of BM MSCs for younger (<30 years of age) and elderly (>70 years of age) individuals showed increased expression of genes associated with mTOR signalling [123]. Studies of both murine and human BM MSCs have shown that miR-188 regulates the age-related switch between osteoblast and adipocyte differentiation, and miR-188 then targets rictor [116]. Animal studies have shown decreased BM levels of IGF-1 in aged rats; IGF-1 seems to stimulate osteoblastic MSC differentiation through activation of mTOR and aging of MSCs may thus be caused by decreased mTOR signalling [131].
**Metabolic Regulation**
MSC proliferation is regulated by extracellular glucose levels; this effect is mediated through both the PKC-MAPK and PI3K-Akt-mTOR pathways [124]. Glycogen synthase kinase 3 β is a metabolic regulator; inactivation of this regulator can be caused by signalling through mTORC2 and Akt phosphorylation at S437 [104].
**Differentiation of MSCs—General Effects**
Murine studies suggest that absence of mTORC1 causes reduced capacity of adipocyte differentiation, whereas absence of mTOCR2 causes reduced osteogenic differentiation capacity and accelerated adipogenesis [91,126]. mTORC2 regulates mechanically induced cytoskeletal reorganization (actin stress fibre development) and favours osteogenesis over adipogenesis [126]. The stemness marker CD49f identifies a subset with high proliferative ability and differentiation potential; downregulation of this marker (i.e., knockdown, tumour necrosis factor α, TNF-α, treatment) is associated with decreased differentiation and downregulation through TNF-α is mediated by mTOR [132].
**Osteogenic Differentiation of MSCs**
IGF-1-induced growth enhancement and osteoblastic differentiation of MSCs is inhibited by mTORC1 inhibitor rapamycin; this IGF-1effect is seen for MSCs derived from different tissues including BM [106,131]. The osteogenic effect of erythropoietin is mediated through various intracellular pathways, including signalling through PI3K and mTOR [113,125]. There seems to be a time-dependent modulation of AMPK-Akt-mTOR signalling during osteogenic differentiation with early activation of AMPK/raptor and thereby mTOR/S6K1 inhibition, and later activation of Akt/mTOR [120]. Stat3 activation seems to be a negative regulator of osteogenic differentiation [115]. BMP-2 and -4 stimulate osteogenic differentiation; JAK2 signalling then mediates Stat3 tyrosine phosphorylation whereas serine phosphorylation is mediated through ERK1/2 and mTOR signalling. Stat3 knockdown accelerates and augments osteogenic differentiation. mTOR inhibitors can increase osteogenic differentiation [119], and studies in human BM MSCs suggest that osteopenia can be induced through PI3K-Akt-mTOR and activation of S6K1 [118]. However, the effects of the PI3K-Akt-mTOR pathway are complex and effects of pathway inhibitors are difficult to predict. Induction of osteogenic differentiation has not been detected in all experimental models, and a possible explanation is that the final effect of mTOR inhibitors depends on the experimental model and the biological context [2]. However, the dual PI3K/mTOR inhibitor BEZ235 strongly inhibited osteogenic differentiation in human MSCs [119].
**Adipocytic Differentiation of MSCs**
Adipocytic differentiation is associated with downregulation of Notch gene expression; modulation of PTEN-PI3K-Akt-mTOR signalling seems important for this Notch effect [127]. Insulin, Akt and mTOR signalling is important in adipocyte differentiation and rapamycin can reduce the expression of most adipocyte markers [2]. mTOR is essential for adipocytes to sense nutrient availability and modulation of PPAR-γ activity that is an important regulator of the adipogenic gene expression program [2]. Differentiation of brown adipocytes requires signalling pathways distinct from white adipocytes; mTOR activity is involved in the initial steps but later inhibition through AMPK activation is also necessary [2].
**Myogenic Differentiation**
A recent review concluded that several studies suggest that mTOR is indispensable for myogenesis, but the mechanisms behind this function are largely unknown [2].
**Regulation of Autophagy and Senescence in MSCs**
Autophagy is the natural regulated mechanism that disassembles unnecessary or dysfunctional cellular components. Cellular senescence is the phenomenon by which normal diploid cells cease to divide, but they remain metabolically active and commonly adopt an immunogenic phenotype consisting of a pro-inflammatory secretome. These two processes seem to be regulated by overlapping mechanisms. AMPK is a positive regulator of autophagy in MSCs; autophagy can then be activated through the AMPK-mTOR pathway and protect BM MSCs from stress-induced apoptosis [133]. Animal studies suggest that senescent BM MSCs show upregulation of p53 and downregulation of mTOR. Knockdown of p53 then alleviates senescence, reduces autophagy and upregulates mTOR [134]. Human BM MSCs show downregulation of Notch gene expression during adipocyte differentiation; Notch inhibition will also enhance adipocyte differentiation and at the same time induce autophagy by acting on the PTEN-PI3K-Akt-mTOR pathway [127]. mTOR inhibition can also reverse the senescent phenotype of human MSCs [110]. Thus, the PI3K-Akt-mTOR pathway is involved in the regulation of senescence/autophagy/apoptosis in BM MSCs.
**Adaptation to the Hypoxic BM Microenvironment**
The BM microenvironment is hypoxic [105,107,111]; the hypoxia seems to causes upregulation of hypoxia-inducible factor 1α (HIF-1α) in primary human AML cells and increased constitutive release of several cytokines by the leukemic cells [112]. Hypoxia induces autophagy and eventually apoptosis in BM MSCs; at the same time hypoxia seems to activate AMPK-mTOR signalling and inhibition of mTOR will then further increase the hypoxia-induced apoptosis [134]. However, hypoxia stimulated by Toll-like receptor (TLR) ligation show decreased apoptosis in response to hypoxia and at the same time increased autophagy and activated AMPK-mTOR signalling [117]. Furthermore, downregulation of leptin will attenuate hypoxia-induced autophagy [128]. Finally, hypoxia also increases the levels of fatty acid synthetase in umbilical cord MSCs; increased signalling through HIF-1α-fatty acid synthase-mTORC1 then represents an important link between hypoxia-induced lipid metabolism and increased proliferation as well as migration of MSCs [114]. Thus, autophagy seems to protect MSCs against hypoxia-induced apoptosis; AMPK-mTOR signalling seems important for regulation of autophagy and thereby also for the adaptation of MSCs to a hypoxic BM microenvironment together with leptin and possibly p53 (see above).
**Communication between MSCs and Neighbouring Cells**
**Fibroblasts:** Fibroblasts can support AML cell proliferation; they also show constitutive release of leukaemia-supporting/angioregulatory cytokines and this release can be altered by PI3K/mTOR inhibition [76]. Conditioned medium from cultures of BM MSCs suppresses fibroblast proliferation; this effect is mediated mainly by TGF-β3 [130]. However, MSCs release a wide range of soluble mediators and other forms of TGF-β that signal through the same receptors [108]; probably, other cytokines/chemokines may also contribute to this effect. PI3K-Akt-mTOR signalling is a downstream effect to TGF-receptors, and this pathway is also important for fibroblast proliferation (both mTORC1 and mTORC2), adherence and release of extracellular matrix molecules [108,121,122,129]. Thus, PI3K-Akt-mTOR targeting may alter this crosstalk between MSCs and fibroblasts both through effects on MSCs and the fibroblasts. **Osteoblasts:** mTOR/S6K1 signalling is important for osteoblast responses to exogenous cytokines and for the regulation of osteoblast cytokine release [2], including cytokines that can support leukemogenesis and modulate other stromal cells including MSCs [2].

**Table 4:** Cytokines released by MSCs and malignant cells.

Soluble Mediators Released by MSCs
**Interleukins**
IL-1α/β [47], IL-6 [55,136], IL-10 [139,140], IL-15 [141]
**Chemokines**
CCL2 [94,95], CCL3 [94], CCL4 [95], CCL5 [94,95], CCL7 [53], CCL20 [95], CCL26 [53], CXCL1 [53], CXCL2 [53], CXCL5 [53], CXCL8 [94,136], CXCL10 [53], CXCL11 [53], CXCL12 [94,95], CX_3_CL1 [95]
**Growth factors**
Ang-1 [94], VEGF [94,136], TGF-β [139,140,142], PDGF [50], bFGF [50], FGF7 [50], HGF [139,140,142], IGF-1 [45], EGF [45], G-CSF [55], M-CSF [55], GM-CSF [55], SCF [55], LIF [55], IFN-β [136]
**Other mediators**
PGE_2_ [139,140,142]
**Soluble Mediators Commonly Released by Various Malignant Cells**
IL-6 [93,143], Ang-1 [144], VEGF [144], TGF-β, BMP-4 [143], Wnt5α [144], Gremlin-1 [144], bFGF [143,144], HGF [143], IGF-I/II [143], EGF [144], CTGF [143], G-CSF [144], CCL5 [93,143], CXCL12 [93,143]

Abbreviations: IL: interleukin; Ang-1: angiopoietin 1; G/M-CSF: granulocyte/macrophage-colony-stimulating factor; LIF: leukaemia inhibitory factor; PGE_2_: prostaglandin E2; CTGF: connective tissue growth factor.

**Table 5:** Important effects of MSC on immunocompetent cells.

**T Cells**
T-cell suppression may be induced by TGF-β, HGF, PGE_2_, IL-10, nitric oxide (NO) or the enzymes indoleamine-2,3-dioxygenase (IDO) and haeme oxygenases (HOs) [68,80,93,139,140,141,142]. PGE_2_ and IDO seem to have the strongest effects [88]: PGE_2_ forces macrophages to produce more IL-10, and enhances the effect of IDO [45], whereas IDO inhibits proliferation through tryptophan degradation [88]. The HO-1 effect is mediated through IL-10 and NO and is also regulated by IDO [139]. NO is produced during chondrogenesis and inhibits Stat5 phosphorylation [142]. The effects of TGF-β and IDO on T-cells are irreversible, whereas T-cell function can be restored after PGE_2_ exposure [142]. MSCs also produce other potentially immunosuppressive factors, like IFN-β [136] and IL-1α/β [47]. MSCs increase the number and trigger the activation of regulatory T-cells (Tregs) [51,149].
**NK Cells**
Secretion of TGF-β, IL-15, PGE_2_ and IDO inhibits cytokine expression, proliferation and cytotoxicity of resting NK cells [53,88,136,137,140,141]. The final effect of MSCs on NK cells may depend on the cellular microenvironment because other studies have described NK cell activation by MSCs [148].
**B Cells**
The effect of MSCs on B-cells is still controversial [151], but MSCs seem to inhibit B-cell proliferation [80,146,147] and modulate their function [140] through cell cycle arrest during G_0_ and G_1_ phases [88,146]. MSCs also seem to inhibit B-cell differentiation, chemokine receptor expression and chemotaxis [146] through soluble factors, e.g., PGE_2_ [53] and CCL2 [145], and cell-cell contact [45]. MSCs have also been linked to enhanced B-cell differentiation [71]. B-cell suppression may at least play an indirect role on T-cell suppression [47,136,150].
**Dendritic Cells**
MSCs influence the differentiation of antigen-presenting DCs [53,78] by inhibiting DC maturation from monocytes [88,141]. MSCs also affect their activation by TLRs [152], their antigen presenting function [146] and their anti-inflammatory potential [53].

**Table 6:** Effects on monocyte differentiation of knocking out single mediators involved in PI3K-Akt-mTOR signalling; the results from animal knockout models.

Media-TOR	M1/M2 Ratio	Comment	Reference
**Akt1**	M1↑M2↓	Akt is expressed as the three different isoform Akt1, Akt2 and Akt 3. These isoforms contributes differentially to differentiation of monocytes into the two main forms: M1: Reduced responsiveness to TLR ligands, reduced secretion of IL-1βα, IL-6 and TNF-α. M2: C/EBPβ seems important for M2 differentiation, Stat6 also seems to be involved together with miR-155. The Akt1 isoform facilitates differentiation into the M2 phenotype, and Akt2 downregulation upregulates C/EBPβ. Akt1 is important for Akt-mediated effects on ECs whereas Akt2 is important in insulin signalling.	[174]
**Akt2**	M1↓M2↑	Akt2 induces differentiation in direction of the pro-inflammatory M1 phenotype.	[174,175]
**AMPKα1**	M1↓M2↑	AMPK activation in macrophages results in results in polarization to the anti-inflammatory M2 phenotype. Exposure of macrophages to IL-10 causes AMPK activation, and AMPKα1 is then required for IL-10 activation of PI3K-Akt-mTORC1 and Stat3-mediated anti-inflammatory pathways regulating macrophage polarization.	[171]
**AMPKβ1**	M1↓M2↑	Animal studies demonstrated that AMPKβ1 deficient macrophages are M1-activated, i.e., AMPK seems to differentiate macrophages towards an immunosuppressive M2 phenotype.	[170]
**PTEN**	M1↓M2↑	PTEN is important for the increased release of pro-inflammatory cytokines such as IL-6 by macrophages in response to TLR ligation, and deletion of PTEN then results in diminished inflammatory responses. Furthermore, macrophages isolated from such knockout mice express higher levels of M2 markers, produce lower TNF-α and higher IL-10 levels in response to TLR ligation. Such M2 macrophages also show enhanced Stat3- and Stat6-signalling together with diminished Stat1-signalling pathway activation in response to TLR4 stimulation.	[172,173]
**PDK1**	M1↑M2↓	A major characteristic of mice with myeloid PDK1 knockout is increased tissue infiltration of macrophages with the M1 phenotype; the authors concluded that PDK1 regulates macrophage migration through inhibition of FOXO-1 induced CCR2 expression.	[190]
**Inpp5d**	M2↑	Inositol polyphosphate-5-phosphatase D. The expression of this protein is restricted to haematopoietic cells and it functions as a negative regulator of myeloid cell proliferation and survival. Deficient murine monocytes are more sensitive to IL4-induced induction of the M2 phenotype.	[191]
**TSC1**	M1↑M2↓	Knockout of TSC1 in the myeloid lineage causes constitutive mTORC1 activation with downregulation of Akt signalling that is essential for resistance to M2 polarization and increased responsiveness to pro-inflammatory stimuli. Thus, the effect can at least partly be explained by increased mTORC1 activity with a negative feedback on Akt function. The TSC1 deficient cells show impaired migration and reduced expression of chemokine receptors, including CCR2 and CCR5, phagocytosis and reactive oxygen species production is increased and the effect of the knockdown is partially reversed by mTORC1 inhibitors.	[176,177,178]
**Raptor**	M1↓M2↑	Raptor deficiency reduced inflammatory gene expression in macrophages derived from several organs, including BM macrophages. This seems to be caused by attenuation of Akt inactivation and increased NFκB signalling.	[192]
**Rictor**	M1↑M2↓	Primary macrophages isolated from myeloid-specific rictor null mice exhibited an exaggerated response to TLRs ligands, and expressed high levels of M1 genes and lower levels of M2 markers.	[179]

**Table 7:** The effect of mTOR inhibition on various biological characteristics of human monocytes/macrophages.

Hallmark	Drug	Cells	Effect	Reference
**Cytokine Production**	Sirolimus	THP-1 human AML monocytic cell line Normal human monocytes	Sirolimus reduced release of CCL2, CCL3, CCL5 and CXCL8 in both human and murine monocytes; CCL4 was in addition reduced in human cells. There was no effect on TNF-α release.	[183]
	Everolimus	C57BL/6 murine cells Normal human monocytes	While mTOR inhibition did not lead to any changes during starvation, everolimus significantly increased production of IL-6, CCL2, CCL5 and TNF-α and except for CCL2 this increase was inhibited by MAPK inhibition.	[184]
	Rapamycin	Normal human monocytes tested in vivo and in vitro	Rapamycin induced apoptosis of M2- but not M1 polarized cells. M1 polarized: rapamycin reduced the release of CXCR4 and expression of CD206 and CD209; also reduced stem cell growth factor β, CCL4 and CCL13. M2 polarized: rapamycin increased expression of CD86, CCR7, IL-6 and TNF-α; reduced CD206, IL-10, VEGF and CCL18. Stimulation by LPS, Listeria Pam3Cys or flagellin; increased release of IL12p40 and IL-23, reduced TNF-α and IL-6.	[185]
	Everolimus	Rat monocytes, in vivo studies	Histological examination of induced experimental neuritis showed that everolimus significantly (i) increased accumulation of M2 cells, spleen M2 cells were also increased; (ii) mRNA levels of INF-γ and IL-17 were reduced whereas they increased for TGF-β and IL-4; (iii) cytokine protein levels showed reduced IL-1α, IFN-γ and CCL5 but increased IL-10 levels.	[181]
	Rapamycin	Human, in vitro	Rapamycin decreased IL-6 and IL-10 but did not affect TNF-α release after LPS exposure.	[186]
In vivo migration	Everolimus	In vivo and in vitro studies Monocytes	Everolimus reduced migration of macrophages to atherosclerotic plaque in the carotis wall; in vitro studies showed reduced migration towards CCL2, CXCL3, CXCL8, C5a and *N*-formylmethionyl-leucyl-phenylalanine.	[180]
Foam cells formation	Everolimus	THP-1 foam cells	Decreased viability of foam cells, no effect on release of IL-1β, CXCL8, TNF-α but reduced release of CCL2; increase cellular clustering.	[182]
Expression of TLRs	Everolimus	Normal human monocytes, in vitro studies	A significant increase in TLR expression by monocytes was seen in patients with drug eluting stents compared with bare metal stents.	[187]
